# High Post-Capture Survival for Sharks, Rays and Chimaeras Discarded in the Main Shark Fishery of Australia?

**DOI:** 10.1371/journal.pone.0032547

**Published:** 2012-02-27

**Authors:** Matias Braccini, Jay Van Rijn, Lorenz Frick

**Affiliations:** 1 Department of Primary Industries Queenscliff Centre, Marine and Freshwater Fisheries Research Institute, Queenscliff, Victoria, Australia; 2 School of Biological Sciences, Monash University, Clayton, Victoria, Australia; University of Wales Swansea, United Kingdom

## Abstract

Most sharks, rays and chimaeras (chondrichthyans) taken in commercial fisheries are discarded (i.e. returned to the ocean either dead or alive). Quantifying the post-capture survival (PCS) of discarded species is therefore essential for the improved management and conservation of this group. For all chondrichthyans taken in the main shark fishery of Australia, we quantified the immediate PCS of individuals reaching the deck of commercial shark gillnet fishing vessels and applied a risk-based method to semi-quantitatively determine delayed and total PCS. Estimates of immediate, delayed and total PCS were consistent, being very high for the most commonly discarded species (Port Jackson shark, Australian swellshark, and spikey dogfish) and low for the most important commercial species (gummy and school sharks). Increasing gillnet soak time or water temperature significantly decreased PCS. Chondrichthyans with bottom-dwelling habits had the highest PCS whereas those with pelagic habits had the lowest PCS. The risk-based approach can be easily implemented as a standard practice of on-board observing programs, providing a convenient first-step assessment of the PCS of all species taken in commercial fisheries.

## Introduction

Sharks, rays and chimaeras (chondrichthyans) are of high conservation concern due to their relatively high vulnerability to fishing overexploitation, resulting from particular life history traits such as low fecundity, late maturation and high longevity [1]. Several cases of overfished and declining shark stocks (see [2] and [3] for a review) have led to a growing concern about the conservation of chondrichthyans and suggest efforts should be made to improve management [4]. Some shark species are commercially targeted but the majority of chondrichthyans are taken incidentally and subsequently discarded [5]. Discarding rates of chondrichthyans have been increasingly quantified over the past decades (e.g. [6], [7]). However, much less is known about the post-capture survival (PCS, i.e. the probability of surviving the catch, handling and release process) of discards, which could be an important factor contributing to the overall impact of fishing on chondrichthyan populations.

The PCS of chondrichthyans has been estimated for very few species, and mostly for species discarded in bottom trawl fisheries directed at commercially valuable groups, such as teleosts and crustaceans (e.g. [8]–[10]). Fisheries targeted at chondrichthyans (mainly shark species) commonly use gillnet and long line fishing gears, both in Australia [11] and worldwide [5]. Gillnets are size selective (e.g. [12], [13]); hence, a common measure applied in the management of chondrichthyan populations is the use of a specific mesh size to avoid catching certain critical size classes (e.g. large breeding females and juveniles). Given that mesh-size selectivity does not discriminate among species, non-commercial species (including protected and endangered species) are incidentally taken and returned to the water. Another commonly used management measure is the establishment of a Total Allowable Catch (TAC) for certain target species [11], [14], [15]. Such TACs vary among target species so once a vessel reaches the TAC for a particular species, individuals of this species are discarded while the fishers continue fishing for other target species for which the TAC has not yet been reached. In addition, TAC management encourages ‘high grading’ where only the most profitable part of the catch is retained while less valuable individuals (e.g. small sizes) are discarded. All these circumstances result in the discarding of individuals from both commercial and non-commercial species. Quantifying the PCS of all species taken in gillnet fisheries is therefore critical for assessing the extent of fishing impacts.

The few studies that have quantified the PCS of chondrichthyans captured in gillnets focused on commercially important species. Rulifson [16] used cages to monitor the condition of spiny dogfish (*Squalus acanthias*) 48 hours after capture, Hueter et al. [17] used conventional tag-recapture methods on bonnethead (*Sphyrna tiburo*), and blacktip sharks (*Carcharhinus limbatus*), and Manire et al. [18] used several blood constituents (e.g. glucose, sodium, lactate) of bonnethead, blacktip and bull (*C. leucas*) sharks as proxies for PCS. Despite some limitations [19], these methodologies are promising and complement each other, but they are also very cost- and labour-intensive. The application of these methods is therefore not a viable option for estimating the PCS of all shark, ray and chimaera species taken in a fishery when research budgets are very limited, especially in developing countries where the largest proportion of sharks and rays is actually captured [5], or when management objectives are focused mainly on the monitoring and assessment of commercially important species. In this context, alternative methods are required to make research and management more cost-effective and priority driven. These methods should build on the best available information, assess a broad range of species simultaneously, be inexpensive, and simple to use; for example, methods that can be incorporated on a routine basis as part of onboard observer monitoring programs.

Observer programs are particularly valuable for addressing discard-related issues because observers witness the capture of large numbers of chondrichthyans across a broad range of species. Port sampling does not yield information on discards, and it is not in the fisher's interest to report such information in log books [20]. There are currently onboard observer programs in developed (e.g. [21], [22]) and developing (e.g. [23], [24]) countries. These programs provide an ideal platform for collecting PCS information during commercial fishing operations.

In the present study, we present a risk-based approach for investigating the PCS of chondrichthyans taken in a gillnet fishery. This approach was trialled as part of a scientific survey designed as a standard onboard observing program in the commercial shark gillnet fishery sector of the Southern and Eastern Scalefish and Shark Fishery (SESSF) of south-eastern Australia [7]. This is the most important Australian shark fishery in terms of landings [11] where gummy shark (*Mustelus antarcticus*) is the main target species but >30 other species of sharks, rays and chimaeras are taken and either retained or discarded [6]. Our objectives were to develop a method for the rapid assessment of the PCS of all chondrichthyan species taken in a gillnet fishery, and, for the most abundant species, test the effects of sex, water temperature, depth, net soak time, and body size on PCS.

## Methods

### Ethics Statement

All research was conducted with approval from the Fish Animal Ethics Committee of the Victorian Department of Primary Industries (Permit # DPI Fish AEC Feb07 0021).

### Study area and data collection

**Figure 1:**
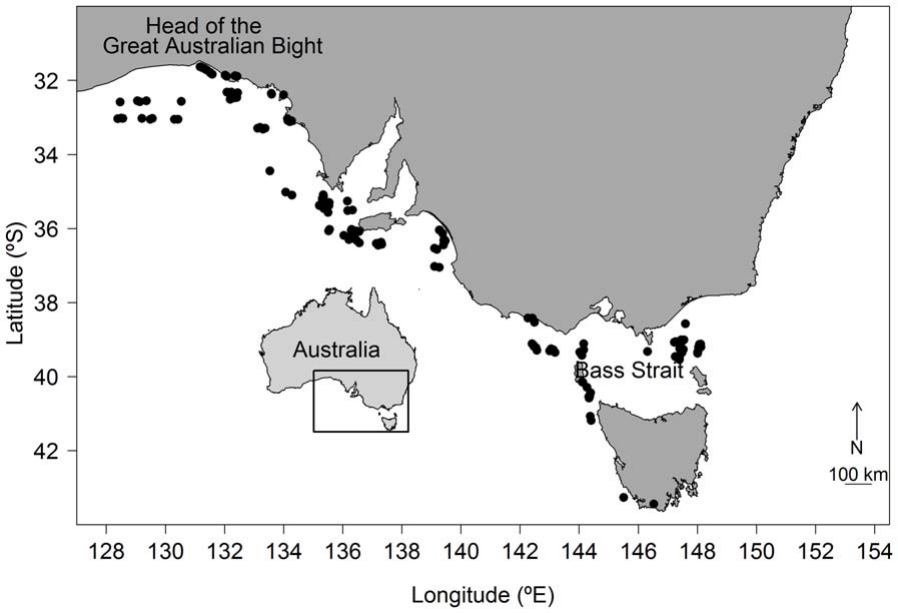
Map of study area. The location of each sampling site along the coast of south-eastern Australia is shown.

Data were collected by onboard observers participating in a fishing survey of the population abundance and size composition of species caught in the SESSF during 2007 and 2008 [7]. The survey was designed to represent fishing practices commonly used in the SESSF. Data were collected on board five commercial shark fishing vessels (15.3–21 m long) using a fleet of demersal monofilament gillnets of five mesh sizes (4, 5, 6, 7, and 8 inches) at 168 sites from eastern Bass Strait to the Head of the Great Australian Bight (Fig. 1). Each net was 500 m long and 2.4 m high, and had a standard hanging coefficient (0.6) and colour (green). Following standard commercial-fishing and onboard-observing practices, sampling was carried out over a variety of habitat types (rocky reefs, bare sand, gravel, mud), times of day (2.00 am–11.30 pm), bottom (12.3–18.7°C) and surface (10.0–21.0°C) temperatures and depths (9–230 m), with net soak times ranging 2.4–20.6 hours. Sharks of different species in the sampling area tend to use waters with different characteristics (e.g. [6]). Our analysis is therefore representative of the PCS of the different chondrichthyan species taken in the fishery. During net hauling, observers recorded the species, sex, and body size (total length for sharks and chimaeras and disc width for rays) of every chondrichthyan captured. In addition, observers collected information used for estimating PCS.

### Immediate, delayed and total post-capture survival

Total PCS was partitioned into an immediate and a delayed component. Immediate PCS was defined as the probability of surviving the capture process prior to discarding. Delayed PCS was defined as the probability of surviving after discarding. Information on physical injury combined with behavioural indices or reflex impairment (i.e. decrease or inhibition of normal behaviour or baseline reflex action) integrate the effects of capture-related stressors by reflecting the status of physiological systems and predatory avoidance mechanisms [25], [26]. Behavioural indices are useful indicators of delayed PCS for bonnethead and blacktip sharks [17]. In this study, we used four categorical indices (Table 1), which reflect physical damage and behavioural condition to predict delayed PCS. The indices ranged 0–1 so delayed PCS also ranged 0–1. The scoring of the indices is simple and rapid, a beneficial characteristic in an onboard observer program. Adopting a precautionary approach, the highest score for a particular value range was used (e.g. 1 for a 0.67–1 value range). The indices were developed combining information from indices previously used for the PCS estimation of sharks [17], [18], from our previous observations made in the field, and from experiments performed with captive sharks under controlled conditions in the laboratories of the Marine and Freshwater Fisheries Research Institute, Victoria, Australia.

**Table 1:** Description of the score values of the indices used for the estimation of PCS for four arbitrary survival categories.

Index	Description	Survival Category			
		High	Moderate	Low	Nil
Activity and stimuli	Physical activity and response to stimuli	1 (strong and lively, flopping around on deck, shark can tightly clench jaws, no stiffness)	0.66 (weaker movement but still lively, response if stimulated or provoked, shark can clench jaws, no stiffness)	0.33 (intermittent movement, physical activity limited to fin ripples or twitches, little response to stimuli, body appears limp but not in rigor mortis, some stiffness)	0 (shark in rigor mortis or dead and limp, stiff and lifeless, no physical activity or response to stimuli, jaws hanging open)
Wounds and bleeding	Presence of wounds and bleeding	1 (no cuts or bleeding observed)	0.66 (1–3 small cuts or lacerations not deep only on skin, some bleeding but not flowing profusely, no exposed or damaged organs)	0.33 (>3 small cuts or one severe cut or wound, some bleeding but not flowing profusely, little organ exposure and if exposed, organs are undamaged)	0 (extensive small cuts or very severe wounds or missing body parts, excessive bleeding, blood flowing freely and continuously in large quantities, internal organs exposed and damaged, may be protruding)
Sea lice	Skin damage by sea lice	1 (no penetration of body by sea lice, body is intact)	0.66 (minor penetration of body by sea lice)	0.33 (moderate body penetration but sea lice mostly on the cloaca area)	0 (extensive penetration of body via eyes, cloaca, gills, and/or skin, sea lice ate tissue)
Skin damage and bruising	Skin damage and surface bruising by physical trauma	1 (0% of skin body damage or bruises or redness)	0.66 (<5% of skin body damage or bruises or redness)	0.33 (5–40% of skin body damage or bruises or redness)	0 (>40% of skin body damage or bruises or redness)

Gummy and Port Jackson (*Heterodontus portusjacksoni*) sharks were caught by a commercial fisher using gillnets in coastal waters of Victoria and were transported to the laboratory in a trailer-mounted fish transport tank containing chilled, aerated seawater. These two species were assumed to represent the range of physiological responses to the catch and release process. Animals were left to acclimatize for at least seven days prior to experimentation in circular, 19,000 L holding tanks connected to a flow-through seawater system running at ambient seawater temperature [27]. Following Frick et al. [27] and Van Rijn [28], one individual at a time was removed from a general population tank and placed in a 5,000 L experimental tank where gillnet capture was simulated. Sharks were manually inserted into a gillnet (3 m long; mesh size 4 or 5 inch, depending on the shark's size), and left there for a period of two hours. Upon removal from the gillnet, each individual was placed in a plastic fish bin with no water for 15 minutes (deck handling simulation), assigned scores for ‘activity and stimuli’, ‘wounds and bleeding’, and ‘skin damage and bruising’ (Table 1), and transferred to a recovery tank where the shark was monitored for ten days.

### Statistical analyses

For estimating immediate PCS, sharks, rays and chimaeras reaching the deck were classified as either dead (‘activity and stimuli’ index = 0, Table 1) or alive (‘activity and stimuli’ index>0, Table 1). The proportion alive was the response variable of a generalised linear model (GLM) used to estimate the immediate PCS of each species. A binomial distribution with a logit link function was used.

Delayed PCS was semi-quantitatively determined following a risk assessment approach (e.g. [29]–[31]). For each individual alive, delayed PCS was calculated as the product of the scores of the four categorical indices. This information was used as the response variable of a GLM with a quasibinomial distribution and logit link function to estimate the delayed PCS of each species.

For each individual, total PCS was calculated as the product of immediate and delayed PCS. This information used as the response variable in a GLM with a quasibinomial distribution and logit link function to estimate the total PCS for each species. Then, a similar GLM model was used to test the effects of sex, depth, body size, net soak time, and water temperature on total PCS. This analysis was only performed for the most abundant species (common sawshark (*Pristiophorus cirratus*), Australian swellshark (*Cephaloscyllium laticeps*), gummy, Port Jackson, and school (*Galeorhinus galeus*) sharks, and spikey dogfish (*Squalus megalops*), Table 2). The opportunistic nature of data collection prevented the modelling of term interactions due to the little contrast in some of the interactions.

**Table 2:** Retention rate of individuals, numbers, delayed mortality descriptors, and predicted immediate, delayed and total PCS.

Common name	Scientific name	Retention		Numbers		Delayed mortality descriptors				Post-capture survival		
		Discarded	Retained	Alive	Dead	Activity and Stimuli	Wounds and bleeding	Sealice	Skin damage and bruising	Immediate	Delayed	Total
Australian angelshark	*Squatina australis*	0.917	0.083	42	14	0.896	0.984	1.000	0.892	0.750	0.798	0.595
Broadnose shark	*Notorynchus cepedianus*	0.146	0.854	135	67	0.726	0.982	0.992	0.847	0.668	0.544	0.335
Bronze whaler	*Carcharhinus brachyurus*	0.010	0.990	97	55	0.705	0.986	0.993	0.901	0.638	0.645	0.408
Cobbler wobbegong	*Sutorectus tentaculatus*	0.000	1.000	6	0	1.000	1.000	0.943	1.000	1.000	0.943	0.943
Common sawshark	*Pristiophorus cirratus*	0.069	0.931	423	139	0.821	0.985	0.994	0.948	0.753	0.707	0.536
Australian swellshark	*Cephaloscyllium laticeps*	1.000	0.000	1968	9	0.980	0.999	1.000	0.999	0.995	0.947	0.939
Elephantfish	*Callorhinchus milii*	0.474	0.526	22	44	0.740	0.949	1.000	0.887	0.333	0.357	0.066
Greenback stingaree	*Urolophus viridis*	1.000	0.000	15	0	1.000	1.000	1.000	0.932	1.000	0.932	0.932
Greeneye dogfish	*Squalus chloroculus*	1.000	0.000	3	2	0.553	1.000	1.000	0.887	0.600	0.516	0.310
Gummy shark	*Mustelus antarcticus*	0.213	0.787	1606	2120	0.784	0.983	0.985	0.877	0.431	0.638	0.257
Ogilby's ghostshark	*Hydrolagus ogilbyi*	0.889	0.111	21	2	0.457	0.936	1.000	0.920	0.913	0.420	0.384
Port Jackson shark	*Heterodontus portusjacksoni*	1.000	0.000	1441	11	0.988	0.998	0.999	0.992	0.992	0.979	0.973
Rusty carpetshark	*Parascyllium ferrugineum*	1.000	0.000	21	3	0.968	1.000	1.000	1.000	0.875	0.948	0.771
School shark	*Galeorhinus galeus*	0.013	0.987	371	990	0.635	0.953	0.991	0.917	0.273	0.506	0.115
Shortfin mako	*Isurus oxyrinchus*	0.400	0.600	5	3	0.528	0.932	1.000	0.664	0.625	0.387	0.242
Smooth hammerhead	*Sphyrna zygaena*	0.064	0.936	13	109	0.588	0.974	1.000	0.948	0.107	0.568	0.061
Southern eagle ray	*Myliobatis australis*	1.000	0.000	126	7	0.965	0.995	0.995	0.958	0.947	0.911	0.854
Southern sawshark	*Pristiophorus nudipinnis*	0.090	0.910	66	47	0.872	1.000	1.000	0.948	0.584	0.758	0.435
Sparsely-spotted stingaree	*Urolophus paucimaculatus*	1.000	0.000	25	1	0.986	1.000	1.000	1.000	0.962	1.000	0.962
Spikey dogfish	*Squalus megalops*	0.999	0.001	1057	121	0.960	0.998	1.000	0.998	0.897	0.949	0.865
Spiny dogfish	*Squalus acanthias*	0.987	0.013	45	7	0.947	0.992	1.000	0.942	0.865	0.900	0.785
Spotted wobbegong	*Orectolobus maculatus*	0.000	1.000	5	0	1.000	1.000	1.000	1.000	1.000	1.000	1.000
Thresher shark	*Alopias vulpinus*	0.429	0.571	3	6	0.663	1.000	0.887	0.663	0.333	0.465	0.175
Varied carpetshark	*Parascyllium variolatum*	1.000	0.000	4	1	0.915	1.000	1.000	0.915	0.800	0.859	0.687
Whiskery shark	*Furgaleus macki*	0.018	0.982	16	207	0.476	0.936	1.000	0.887	0.072	0.368	0.025

To investigate life history patterns in total PCS, all sharks, rays and chimaeras were classified according to their position in the water column as bottom-dwelling, demersal, or pelagic following Compagno [32], [33] and Last and Stevens [34]. Other traits, such as presence and relative size of spiracles, and body form, are highly correlated with water column position so it is not possible to accurately measure their individual effects. Position in water column was therefore used as the best surrogate for life history traits. A GLM was used to test the effects of water column position on total PCS.

## Results

A total of 11,501 individuals from 25 shark, ray and chimaera species were assessed (Table 2). For each species, sample sizes varied according to their natural abundance, availability and gillnet catchability in the studied area. The most abundant species, and hence those for which PCS estimates are more robust, were gummy and school sharks, and common sawshark (which comprised the bulk of the retained catch), and Australian swellshark, Port Jackson shark and spikey dogfish (which comprised the bulk of the discarded catch). Immediate PCS varied from 1.00 for spotted (*Orectolobus maculatus*) and cobbler (*Sutorectus tentaculatus*) wobbegongs, and greenback stingaree (*Urolophus viridis*) to 0.07 for whiskery shark (*Furgaleus macki*) (Table 2).

The ‘activity and stimuli’ index was the most variable descriptor of delayed PCS, ranging from 1.00 for spotted and cobbler wobbegongs, and greenback stingaree to 0.46 for Ogilby's ghostshark (*Hydrolagus ogilbyi*) (Table 2). All species had ‘wounds and bleeding’, and ‘sea lice’ indices values of 1.00 or very close to 1.00. Most species had a ‘skin damage and bruising’ index of 1.00 or close to 1.00 with the exception of shortfin mako (*Isurus oxyrinchus*) and thresher shark (*Alopias vulpinus*) which had a value of 0.66. Delayed PCS varied from 1.00 for spotted wobbegong and greenback stingaree to 0.36 for elephantfish (*Callorhinchus milii*).

**Figure 2:**
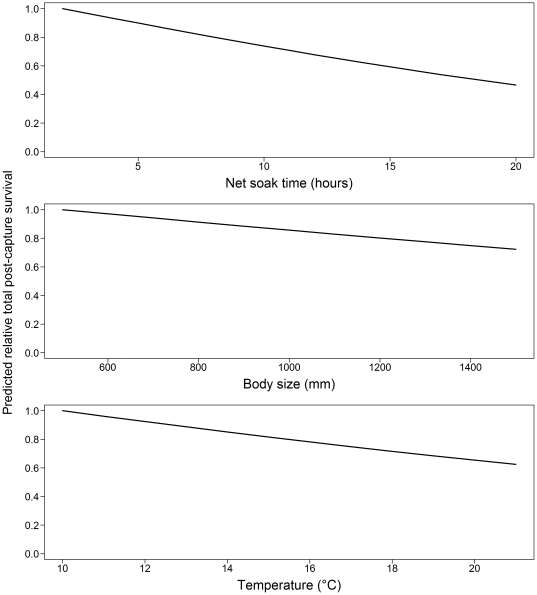
Predicted relative effect of net soak time, body size and temperature on total PCS. The analysis is based on 3224 observations for the six most abundant species.

**Figure 3:**
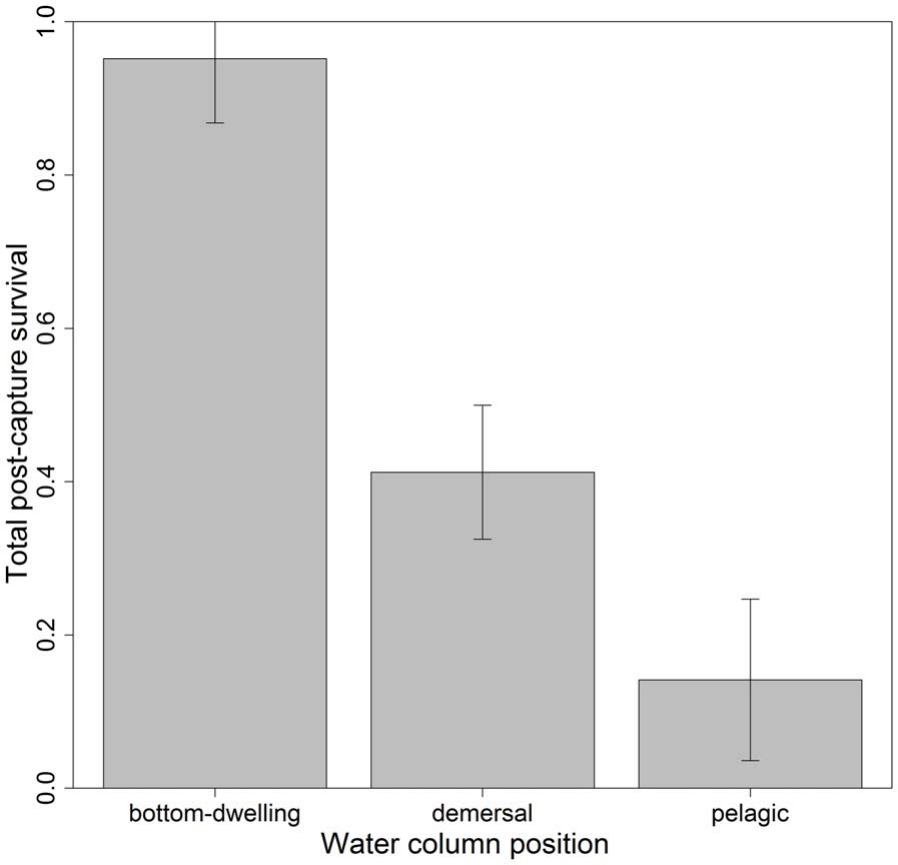
Predicted effect (±SE) of position in the water column on total PCS. The analysis is based on 3065 observations for bottom-dwelling species, 6445 observations for demersal species and 1991 observations for pelagic species.

Of the 25 species assessed, 13 species had a total PCS>0.50, corresponding mostly to discarded species (Table 2). Total PCS varied from 1.00 for spotted wobbegong and greenback stingaree to 0.03 for whiskery shark. Species, net soak time and water temperature had a highly significant effect on total PCS whereas body size had a marginally significant effect. Depth and sex had no effect on total PCS (Table 3). Sharks exposed to the maximum recorded soak time and water temperature had on average 53% and 38% lower total PCS, respectively, than those exposed to the minimum recorded soak time and temperature. The largest individuals had about 27% lower total PCS than the smallest individuals (Fig. 2). Position in water column had a highly significant effect on total PCS (*P*<0.05) (Fig. 3). Bottom-dwelling species had the highest PCS followed by demersal and pelagic species.

**Table 3:** Summary of GLM analysis testing the effects of depth, species, net soak time, body size, water temperature and sex on total PCS.

Terms	Df	Deviance	P
Depth	1	0.337	0.458
Species	5	1642.420	<0.001
Net soak time	1	11.072	<0.001
Body size	1	2.769	0.034
Water temperature	1	9.851	<0.001
Sex	1	0.012	0.890

For the experimental treatment, the estimated total PCS based on the risk assessment method was 1.00 and 0.48 for Port Jackson and gummy sharks, respectively. These estimates showed a strong correlation with the actual survival observed after ten days of monitoring (r = 1.00 for Port Jackson shark and r = 0.89 for gummy shark).

## Discussion

Through the research conducted in this study, we developed a risk assessment method to estimate the PCS of multiple species of sharks, rays, and chimaeras captured in a gillnet fishery. The indices developed were tailored to the specific stressors and consequences associated with being a shark, ray or chimaera caught by gillnet fishing gear and landed on commercial fishing vessels. Our risk-based method aims to provide fisheries scientists and on-board observers with a simple tool for a first-level assessment of the PCS of all chondrichthyan species taken in gillnet fisheries.

### Post-capture survival probability

Over 11,000 individuals from 25 chondrichthyan species were assessed in this study. Only six species (school, gummy, thresher, and whiskery sharks, smooth hammerhead (*Sphyrna zygaena*), and elephantfish) had immediate PCS<0.50 and the majority of discarded individuals had high total PCS, indicating that most of the catch reaching the deck of vessels is alive and likely to survive the initial handling and release process. Other studies on gillnet fishing also showed high values of PCS. For example, bonnethead, blacktip and bull sharks captured in commercial gillnets had >0.60 PCS [17], [18]. The findings reported in the present study are also consistent with controlled studies of survivorship following commercial gillnet capture and handling. Laboratory studies of physiological stress and PCS on representative species from vulnerable (gummy shark) and robust (Port Jackson shark and Australian swellshark) species, showed that gummy shark had a much lower (0.30) PCS than Port Jackson shark (1.00) and Australian swellshark (0.98) [27], [28], [35], [36]. These laboratory findings match very closely the PCS estimates of gummy (0.26), Port Jackson shark (0.97) and Australian swellshark (0.94) based on the risk assessment approach.

Gear type and exposure time have significant effects on PCS. For example, spiny dogfish caught in gillnets but exposed to longer soak times (19–24 hours), showed lower values of PCS (0.45) [16] than in our study whereas shortfin mako, common thresher and blue (*Prionace glauca*) sharks had 0.74, 0.99 and 0.95 PCS, respectively, after three-hour exposure to longline gear [37]. Our observed lower PCS values for shortfin mako (0.63) and common thresher shark (0.33) are likely a result of the longer soak times and the use of gillnets. In this gear, swimming capacity and thus ventilation, particularly for pelagic obligate ram-ventilating species such as shortfin mako and thresher shark, is much more restricted than in long line gear where captured individuals can continue to swim.

Species with pelagic habits (e.g. school and mako sharks) had considerably lower PCS (0.14±0.10) than bottom-dwelling (e.g. Port Jackson shark and Australian swellshark) species (0.94±0.08). In addition, increased soak time and water temperature significantly decreased the PCS of chondrichthyans (e.g. [38], present study) and of other groups (e.g. [25], [39], [40]). These patterns are attributed to differences in the metabolic rate of the different species studied. Metabolic rate can be used to explain patterns in active and non-active shark species [41], with species-specific differences in gillnet survival being associated to respiratory physiology and the degree of struggling upon capture [18]. For example, highly active species, such as pelagic sharks, may initially struggle more vigorously to escape the net, causing individuals to become more tightly enmeshed and exhausted; hence, as they generally depend on ram-jet ventilation for respiration [2], PCS decreases. Furthermore, species with higher anaerobic capacity are expected to have lower PCS due to a higher metabolic acid load triggered by capture stress, and the resulting disruption of the acid-base balance [18], [37], [42]. Finally, pelagic species rely on movement for ventilation and have a fusiform body form which increases the chances of passing their heads through the meshes and becoming even more enmeshed. Bottom-dwelling species, on the contrary, are generally more sluggish, and the presence of spiracles allows them to maintain gill ventilation even if they are constrained. They therefore do not face an immediate risk of asphyxiation, which may be why bottom-dwelling sharks generally fight less once enmeshed (e.g. Port Jackson shark and Australian swellshark) compared with pelagic species (J.M. Braccini personal observation). A reduced struggling effort means reduced metabolic activity, which in turn results in a reduced accumulation of harmful metabolic by-products and thus an increased chance of survival [42], [43].

### Justification of method, limitations and future directions

As in any risk assessment, the methodology presented in this study is a first step to indentifying which species are more at risk. It provides an alternative and demonstrates that we are able to gain comparable knowledge on PCS for a large number of species from observations conducted on board commercial fishing vessels. All species showed little variation in the ‘wounds and bleeding’ and ‘sea lice’ indices values, suggesting that these indices could be omitted from future assessments under similar conditions. On the contrary, the ‘activity and stimuli’ index would be a cost-effective method for assessing the general condition of an animal in order to predict subsequent events in its life. For example, release condition (an index comparable to the activity and stimuli' index) was one of the best and most consistent predictors of the PCS of tropical reef fish and at the same time simple enough to be used by recreational fishers for a broad assessment of species [44].

Quantifying the PCS of chondrichthyans is associated with considerable logistical challenges. Nonetheless, a number of studies have addressed this topic using a range of approaches: tag recapture experiments (e.g. [38]), onboard or water cages [8], [45], [46], replication of fisheries capture in controlled settings [36], [47], and acoustic or satellite tracking of captured and released individuals (e.g. [48]–[50]). In addition, there have been various attempts to predict PCS based on physiological indicators of stress (e.g. [18], [37], [42]). The risk assessment approach is not intended to replace these more rigorous methods. Though these methods provide great insight into a species ability to cope with capture stress, they are very time consuming and expensive, limiting the possibility of conducting a broad assessment of the PCS of all discarded species, particularly when research resources are very limited. These methods can be used to validate the estimates obtained from a risk assessment approach. For example, stress physiology experiments [27], [28], [35], [36] and the risk assessment approach produced similar PCS estimates for vulnerable (gummy shark) and robust (Port Jackson shark and Australian swellshark) species, providing promising support of the fieldwork risk assessment approach. However, activity and condition indices vary with species [51] so further refinement and tuning of the indices used is required.

Our PCS estimates are based on the assumption that deck handling time is kept to a minimum (i.e. individuals are quickly returned to the water after removal from the net). However, individuals from Port Jackson shark, Australian swellshark and Southern eagle ray (*Myliobatis australis*) can be left on deck for several hours before returning to the water. Furthermore, fishers may ‘strike’ individuals of these species on the head to reduce on board thrashing prior to discarding (J.M. Braccini personal observation). The combination of extended time out of water and deliberate mistreatment of discards is likely to decrease PCS and needs to be quantified to obtain more accurate estimates. Several other potential factors not quantified in this study (e.g. sun exposure, humidity, sea condition, or pressure change) that may affect PCS should also be considered.

### Conclusions and conservation remarks

A very large proportion of chondrichthyan global catches is discarded [5], [52] though little is known about the fate of discarded individuals. Hence, PCS information is rarely considered as part of the strategies addressing the management of discarded chondrichthyan species. Given that chondrichthyans remain a low priority for fishery management agencies in general, cost- and labour-intensive research on the broad range of species taken in commercial fisheries may not be conducted in the short term. However, the current change in natural resource management objectives from single-species to ecosystem-wide objectives warrants a multi-species assessment of PCS. Yet multi-species assessments are more difficult, and finding more cost-effective and priority driven methods is important because chondrichthyans continue to be depleted and time and funding for comprehensive data collection is limited [30]. Our study provided species-specific estimates of PCS, showing that these estimates varied among species, but they were generally high for most discarded species. The risk-assessment approach is simple and easy to implement in the onboard observer programs currently monitoring commercial fisheries around the globe, allowing the identification of species of conservation concern, and the prioritization and better direction of research and conservation effort.
